# Surgical Strategies in the Era of SARS-CoV-2

**DOI:** 10.11604/pamj.2020.35.2.22975

**Published:** 2020-04-27

**Authors:** Dimitrios Dimitroulis, Nikolaos Garmpis, Christos Damaskos

**Affiliations:** 1 Second Department of Propedeutic Surgery, Laiko General Hospital, Medical School, National and Kapodistrian University of Athens, Athens, Greece

**Keywords:** COVID-19, surgeries, transplantations

## To the editors of Pan African Medical Journal

The unique and phenomenal pandemic of Severe Acute Respiratory Syndrome Coronavirus 2 (SARS-CoV-2) is monopolizing medical literature, political conversation and social behavior. The unknown course of the infection raises questions regarding its epidemiology, potential treatment modalities and mostly prevention strategies that vary from herd immunity to general lockdown of all financial and social activities. These strict protocols are affecting all aspects of our lives worldwide and disrupting the normal function of public and private hospitals that are preparing for this emerging and rapidly evolving situation. Thus, surgical service is being directed towards emergency situations only and elective surgical procedures are being cancelled or postponed.

An extended part of surgical interventions relates to malignant diseases. The ideal time interval from diagnosis to surgical treatment for malignancies depends on medical data and management capacity of the several health systems. However, it is generally accepted that timing is critical for early-stage malignancies where initial surgical resection is the treatment of choice. The pertinent literature concludes that the sooner an oncological patient undergoes the initial operation, the better the survival chances are. A classic example is early stage colo-rectal cancer where the ideal timing for definitive resection is three to six weeks after diagnosis [1]. Several references regarding other malignancies such as gastric cancer, breast cancer or lung cancer conclude similar results for the time interval between initial diagnosis and surgical resection. Furthermore, new combined treatment modalities for more advanced cases of gastrointestinal and pancreatic tumors include initial administration of pre-operative chemotherapy and/or radiotherapy followed by surgical resection in specific time after chemo/radiotherapy. Delayed delivery of surgical intervention leads to unfavourable outcomes [2]. The epidemiology of malignancies is also a matter of consideration in the management of public health issues. For example, gastric cancer is the fifth most common cancer worldwide and the third cancer related cause of death [3] while colorectal cancer kills more than 700,000 people every year making it the world´s fourth most deadly cancer [4].

Beside malignant diseases, a further major issue in the SARS-CoV-2 era is the limitation or cancelling of transplantation procedures. The treatment of choice for the end stage disease of abdominal and endothoracic viscera is transplantation. Even in the cases of renal failure, kidney transplantation offers a longer life span and an improved quality of life compared to any other treatment option like hemodialysis. For liver, heart and lung failures, any other strategy serves more as a bridge to transplantation than as treatment modality. The lack of grafts, for the rising number of patients in need for a graft, has urged the transplantation community to expand donor criteria and establish living donation. The cancelation of transplantations, even from a living donor, creates an increase in the discrepancy between patients on the waiting list and those who receive a transplantation resulting in an increase in the mortality and morbidity of these patients [5].

## Conclusion

The unprecedented spread of the pandemic has inhibited the usual function of hospitals affecting surgical procedures in order to prepare the system for a major number of infected patients who would need hospitalization or intubation and an intensive care unit bed. However, an effort could be made in keeping the number of oncological surgical interventions to a minimum, especially for patients with intention to treat interventions and those on transplantation the waiting lists. On the other hand, measures to eliminate virus spread during surgery are necessary. Open surgery over laparoscopic procedures is preferred, as gas aspiration during laparoscopic surgeries is considered as a possible way to spread viral diseases. It is a crucial time and the need to minimize both the somatic and psychological imprint of this pandemic over the next few days is urgent.

## Competing interests

All authors declare no competing interests.
